# Short-term inspiratory muscle training potentiates the benefits of aerobic and resistance training in patients undergoing CABG in phase II cardiac rehabilitation program

**DOI:** 10.5935/1678-9741.20150043

**Published:** 2015

**Authors:** Bárbara Maria Hermes, Dannuey Machado Cardoso, Tiago José Nardi Gomes, Tamires Daros dos Santos, Marília Severo Vicente, Sérgio Nunes Pereira, Viviane Acunha Barbosa, Isabella Martins de Albuquerque

**Affiliations:** 1 Universidade Federal de Santa Maria (UFSM), Santa Maria, RS, Brazil.; 2 Department of Physiotherapy. Universidade de Santa Cruz do Sul (UNISC), Santa Cruz do Sul, RS, Brazil.; 3 Department of Physiotherapy. Centro Universitário Franciscano (UNIFRA), Santa Maria, RS, Brazil.; 4 Cardiac Rehabilitation Program. Hospital Universitário de Santa Maria (HUSM), Santa Maria, RF, Brazil and Universidade Federal de Santa Maria (UFSM), Santa Maria, RS, Brazil.; 5 Department of Physiotherapy and Rehabilitation. Universidade Federal de Santa Maria (UFSM), Santa Maria, RS, Brazil.

**Keywords:** Myocardial Revascularization, Rehabilitation, Exercise, Breathing Exercises

## Abstract

**Objective:**

To investigate the efficiency of short-term inspiratory muscle training program associated with combined aerobic and resistance exercise on respiratory muscle strength, functional capacity and quality of life in patients who underwent coronary artery bypass and are in the phase II cardiac rehabilitation program.

**Methods:**

A prospective, quasi-experimental study with 24 patients who underwent coronary artery bypass and were randomly assigned to two groups in the Phase II cardiac rehabilitation program: inspiratory muscle training program associated with combined training (aerobic and resistance) group (GCR + IMT, n=12) and combined training with respiratory exercises group (GCR, n=12), over a period of 12 weeks, with two sessions per week. Before and after intervention, the following measurements were obtained: maximal inspiratory and expiratory pressures (PImax and PEmax), peak oxygen consumption (peak VO_2_) and quality of life scores. Data were compared between pre- and post-intervention at baseline and the variation between the pre- and post-phase II cardiac rehabilitation program using the Student's t-test, except the categorical variables, which were compared using the Chi-square test. Values of *P*<0.05 were considered statistically significant.

**Results:**

Compared to GCR, the GCR + IMT group showed larger increments in PImax (*P*<0.001), PEmax (*P*<0.001), peak VO_2_ (*P*<0.001) and quality of life scores (*P*<0.001).

**Conclusion:**

The present study demonstrated that the addition of inspiratory muscle training, even when applied for a short period, may potentiate the effects of combined aerobic and resistance training, becoming a simple and inexpensive strategy for patients who underwent coronary artery bypass and are in phase II cardiac rehabilitation.

## Abstract (pt)

**Objetivo:**

Avaliar os efeitos de um programa de treinamento muscular inspiratório em curto prazo associado ao exercício aeróbio e resistido na força muscular respiratória, capacidade funcional e qualidade de vida de pacientes submetidos à cirurgia de revascularização do miocárdio, na Fase II da Reabilitação Cardíaca.

**Métodos:**

Trata-se de um estudo quase-experimental, prospectivo, com amostra de 24 pacientes submetidos à cirurgia de revascularização do miocárdio alocados aleatoriamente para Fase II da Reabilitação Cardíaca em dois grupos: grupo de treinamento muscular inspiratório associado ao treinamento combinado (aeróbio e resistido) (GRC + TMI, n=12) e grupo treinamento combinado e exercícios respiratórios (GRC, n=12) durante um período de 12 semanas, com 2 sessões semanais. Antes e após a intervenção, as seguintes mensurações foram obtidas: pressão inspiratória e expiratória máxima (PImáx e PEmáx), consumo de oxigênio de pico (VO_2_pico) e escore de qualidade de vida. Os dados foram comparados entre o momento pré e pós-intervenção na linha de base e a variação entre o pré e pós-programa de reabilitação cardíaca com uso do teste t de Student, exceto as variáveis categóricas, que foram comparadas pelo teste de qui-quadrado. Foi considerado um *P*<0,05.

**Resultados:**

Comparado ao GRC, o GRC + TMI apresentou maior incremento tanto na PImáx (*P*<0,001) quanto na PEmáx (*P*<0,001), no VO_2_pico (*P*<0,001) e na qualidade de vida (*P*<0,001).

**Conclusão:**

Demonstrou-se que a adição do programa de treinamento muscular inspiratório, mesmo quando aplicada por um curto período, pode complementar os efeitos do exercício aeróbio combinado ao resistido, tornando-se uma estratégia benéfica para pacientes submetidos à cirurgia de revascularização do miocárdio na Fase II da reabilitação cardíaca.

**Table:** 

**Abbreviations, acronyms & symbols**	
CABG	Coronary artery bypass
CAD	Coronary artery disease
CRP	Cardiac rehabilitation program
GCR	Group of cardiac rehabilitation
IMT	Inspiratory muscle training
Peak VO_2_	Peak oxygen consumption
PEmax	Maximal expiratory pressure
PImax	Maximal inspiratory pressure
RMS	Respiratory muscle strength

## INTRODUCTION

Cardiovascular diseases are the leading cause of death and disability in Brazil and worldwide. According to the World Health Organization, 7.3 million deaths worldwide were due to coronary artery disease (CAD) in 2008^[1]^. According to Datasus, in Brazil in 2009 there were 209,029 hospital admissions due to CAD, totaling 12,619 deaths with a mortality rate of 6.04%^[2]^.

Despite advances in clinical therapy and percutaneous interventions, coronary artery bypass grafting (CABG) is still widely used in the treatment of patients with CAD because it can control persistent ischemia and its progression to acute myocardial infarction, as well as provide symptomatic relief and prevent ischemic complications^[3]^. However, cardiac surgery is a complex procedure that triggers major organ repercussions, which changes the physiology of patients in many ways^[4]^.

In this sense, it has been suggested that respiratory muscle dysfunctions associated with decreased functional capacity contribute to the prolonged period of lung function recovery and the occurrence of physical deconditioning, which can last several weeks in patients submitted to CABG^[5,6]^.

Several studies have demonstrated the effectiveness of inspiratory muscle training (IMT) in restoration of ventilatory function, decrease in the length of hospital stay, and improvement of functional capacity and quality of life (QoL) of patients who underwent CABG and are in phase I cardiac rehabilitation program (CRP)^[7-10]^. Onishi et al.^[11]^ found that the inclusion of resistance training combined with aerobic training for six months during phase II CRP was beneficial to patients with metabolic syndrome submitted to CABG. However, the short-term effects of IMT in patients in phase II CRP after CABG and its association with aerobic and resistance training have been largely unexplored in the literature and require further elucidation. Therefore, the aim of this study was to investigate the efficiency of short-term IMT associated with combined aerobic and resistance training on respiratory muscle strength (RMS), functional capacity, and QoL in patients who underwent CABG and are in phase II CRP.

## METHODS

### Study design

A prospective quasi-experimental study was conducted among patients who underwent CABG and were recruited from the waiting list for a phase II CRP at the Outpatient Cardiology Clinic of Hospital Universitário de Santa Maria (HUSM), Santa Maria, RS, Brazil. The eligibility criteria included patients undergoing CABG up to three weeks before the initiation of the study at HUSM, a clinical course without complications during hospitalization, the absence of smoking (previous or current), and agreement to participate. Patients with chronic obstructive pulmonary disease, unstable angina, acute decompensated heart failure, acute pericarditis or myocarditis, complex arrhythmias, uncontrolled hypertension, severe orthopedic or neurological disorders, uncontrolled diabetes, and labyrinthitis were excluded.

The study was approved by the Research Ethics Committee of Universidade Federal de Santa Maria (UFSM) under protocol no. 16149813.3.0000.5346 and was conducted in accordance with the Guidelines and Norms Regulating Research Involving Humans established by Resolution no. 466/2012 of the National Health Council.

### Patients and Intervention

Patients eligible for the study were initially assessed via anamnesis, physical examination, and evaluation of inspiratory muscle strength. Subsequently, these patients were randomly allocated to phase II of the CRP into two groups: a group subjected to CRP+IMT (GCR+IMT) followed the IMT protocol in addition to the combined training (aerobic and resistance training) and a group subjected to CRP (GCR) followed the combined training protocol and performed breathing exercises for 12 weeks. An RMS test was conducted before and after the intervention, and the functional capacity and QoL were evaluated. All evaluations were conducted by investigators blinded to the allocation of patients into the intervention groups.

### Cardiac Rehabilitation Program

All patients participated in the CRP for a period of 12 weeks, with two sessions per week (24 sessions). Each session lasted 60 minutes, and all sessions were under the direct supervision of a physical therapist. The training program consisted of a combination of aerobic and resistance exercises, 30 minutes of aerobic exercise on a treadmill and exercise bike, 20 minutes of resistance exercises for the arms (latissimus dorsal m.,biceps brachii m., triceps brachii m., deltoid m., trapezius m., pectoralis major m., pectoralis major m., and rhomboid m) and legs (femoral quadriceps m., hip adductors m. and hip abductors m.) with dumbbells, ankle weights, or elastic bands (3 sets of exercises for each muscle group performed with 10 repetitions with the intensity adjusted to 50% of the load of one maximum repetition - 1MR), and 10 minutes of stretching and relaxation. Heart rate, blood pressure, and peripheral oxygen saturation were measured at the beginning, during, immediately after, and five minutes after each session.

The exercise intensity was based on the percentage of heart rate reserve, calculated as the difference between the maximum heart rate obtained in the exercise stress test and the resting heart rate, with the establishment of an intensity of 55%-65%^[12]^ and a score of 4-6 on the modified Borg scale (ranging between 0 and 10).

In addition to the CRP, the participants performed diaphragmatic stimulation and fractionated breathing patterns (short inspirations during three intervals with a mild inspiratory pause) to achieve a diaphragmatic breathing pattern similar to that performed by the GCR+IMT.

### Inspiratory Muscle Training

The participants assigned to the GCR+IMT group were subjected to IMT, using the IMT Threshold^®^ equipment (Threshold Inspiratory Muscle Trainer, Health Scan Products Inc., Cedar Grove, NJ, USA) in 3 sets of 10 repetitions with an inspiratory load of 30% of the maximal inspiratory pressure (PImax)^[13]^. During training, the participants remained seated with the nose occluded by a nose clip and were advised to maintain a diaphragmatic breathing pattern and a respiratory rate between 15 and 20 cycles per minute. Each week, the training load was adjusted to maintain 30% of the PImax.

### Assessment of Respiratory Muscle Strength 

PImax and maximal expiratory pressure (PEmax) were measured using a digital manometer (MVD-300, Globalmed, Porto Alegre, RS, Brazil)^[14]^. A 2-mm orifice in the system kept the glottis open and prevented any interference from pressure produced by facial muscles. First, the subjects were instructed to remain in a seated position. A demonstration of how the maneuvers should be carried out was given and then performed by the subject after the placement of a nose clip. The subjects were instructed to keep their lips sealed tightly around the mouthpiece so no air could escape. PImax values were obtained by inspiration from residual volume^[14]^, which was repeated at least three times with a one-minute interval between repetitions. PEmax was obtained by expiration from total lung capacity, using the same methodology applied in inspiration. During the PImax maneuver, the subject kept the mouthpiece in the oral cavity only during the inspiration, and in the PEmax maneuver, only during expiration.

The maneuvers were sustained at maximal force for approximately one second and the highest value was computed from a minimum of three repetitions for each maneuver, with a maximum difference of 10% between values and they were then compared to the predicted values according to the equations proposed by Neder et al.^[15]^.

### Assessment of Functional Capacity

Functional capacity was evaluated by exercise testing (ET) using a standard Bruce protocol and assessed with peak oxygen consumption (peak VO_2_). The values of peak VO_2_ were obtained by use of a treadmill stress test (Imbramed^®^ KT 10200, Sao Paulo, Brazil), and the analysis of peak VO_2_ was carried out using the Ergo PC version 2.2 (Micromed^TM^, Brazil) software. The ET was performed according to the guidelines of the Brazilian Society of Cardiology/Department of Exercise, Ergometry, and Cardiovascular Rehabilitation^[16]^.

### Evaluation of Quality of Life

QoL was assessed with the Portuguese version of the Minnesota Living with Heart Failure Questionnaire (MLwHFQ)^[17]^.

### Sample size calculation

To estimate the sample size, a pilot study was conducted using a protocol identical to that described above in a group of five patients. A sampling error of 2%, a two-sided alpha of 5%, a statistical power of 80%, and a difference of 20.6±9.6 cmH_2_O in variation of PImax between the groups were considered as well as a 10% loss to follow-up, thus resulting in the inclusion of at least nine patients per group.

### Statistical Analysis

Data were analyzed using the statistical software SPSS version 20.0. The normality of the variables was assessed with the Shapiro-Wilk test. Categorical data are presented as absolute frequencies and percentages. Continuous data with normal distribution are expressed as means and standard deviations. Student's t-test for paired samples was used to compare the data before and after the intervention. The baseline data and the variation between pre- and post-CRP values between groups were compared using the independent Student's t-test, except for the categorical variables, which were compared by the Chi-square test. A value of *P*<0.05 was considered statistically significant.

## RESULTS

Of the 28 eligible patients, four were excluded for not meeting the inclusion criteria. Therefore, 24 patients were included in the study. Of these, 12 patients were allocated to the GCR and 12 were allocated to the GCR+IMT. No adverse events were observed during the CRP and adherence to the program was considered excellent.

The demographic, anthropometric, and clinical characteristics of both groups are shown in Table 1. No significant differences were observed between the two groups.

**Table 1:** Demographic and clinical characteristics of patients participating in the study.

Baseline characteristics	GCR (*n*=12)	GCR+IMT (*n*=12)	*P*
Age (years)	59.5±8.7	55.2±7.9	0.313
Male gender, n (%)	10 (83.3)	7 (58.3)	0.178
BMI (Kg/m^2^)	28.4±3.8	30.7±4.4	0.274
Diabetes, n (%)	4 (33.33)	3 (25.0)	0.500
Hypertension, n (%)	4 (33.33)	5 (41.6)	0.312
Ejection fraction (%)	65.2±9.9	63.5±3.6	0.640
NYHA			
I, n (%)	2 (16.7)	3 (25.0)	0.849
II, n (%)	8 (66.7)	6 (50.0)	0.408
III, n (%)	2 (16.7)	3 (25.0)	0.849
Extent of disease (%)			
2-vessel	54.3	50.5	0.285
3-vessel	45.7	49.5	
Extracorporeal circulation, n (%)	12 (100)	12 (100)	-
Duration of hospitalization after surgery (days)	6.7±1.7	7.1±1.5	0.657
Peak VO_2_ (mL.Kg^1^.min^-1^)	26.0±5.6	25.5±3.7	0.802
Peak VO_2_^2^ (% predicted)	88.1±23.0	86.4±16.0	0.836
PImax (c^2^mH_2_O)	72.0±8.1	67.8±9.0	0.782
PImax (% predicted)	71.4±13.8	70.4±11.1	0.856
PEmax (cmH_2_O)	87.3±11.0	86.6±25.9	0.936
PEmax (% predicted)	80.2±13.9	86.1±27.7	0.518
MLwHFQ (score)	39.9±12.6	41.0±19.0	0.871
Medication, n (%)			
Acetylsalicylic acid	8 (66.6)	6 (50.0)	0.408
Clopidogrel or triclopidine	2 (16.7)	1 (8.3)	0.500
Statin	6 (50.0)	6 (50.0)	1.000
Diuretics	4 (33.3)	3 (25.0)	0.500
Warfarin	1 (8.3)	2 (16.7)	0.500
ACEI or ARB	4 (33.3)	5 (41.6)	0.500
Beta-blockers	8 (66.6)	9 (75.0)	1.000

Data are expressed as mean ± standard deviation or absolute values and percentages.

ACEI=angiotensin-converting enzyme inhibitor; ARB=angiotensin II receptor blocker; BMI=body mass index; GCR=cardiac rehabilitation group; GCR+IMT=cardiac rehabilitation group + inspiratory muscle training program; MLwHFQ=Minnesota living with heart failure questionnaire; NYHA=New York Heart Association; Peak VO2=peak oxygen consumption; PEmax=maximal expiratory pressure; PImax=maximal inspiratory pressure

A significant increase in PImax and PEmax was observed after CRP in both groups. However, the variation between pre- and post-CRP values was significantly higher in the GCR+IMT (Table 2).

**Table 2:** Comparison of respiratory muscle strength between groups.

Variables	GCR (n=12)				GCR+IMT (n=12)				
	Pre	Post	Variation	P*	Pre	Post	Variation	*P* *	*P* **
PEmax (cmH_2_O)	87.3±11.0	89.8±10.5	2.3±0.8	0.004	86.6±25.9	108.1 ±17.1	21.5±8.5	<0.001	<0.001
PEmax (% predicted)	80.2±13.9	82.2±15.6	2.5±0.8	0.038	86.1±27.7	108.1±21.1	22.0±7.7	<0.001	<0.001
PImax (cmH_2_O)	72.0±8.3	77.0±5.8	4.9±0.2	<0.001	67.8±9.0	96.7±21.3	28.9±7.9	<0.00	<0.001
PImax (% predicted)	71.4±13.8	76.4±12.8	4.7±0.9	<0.001	70.4±11.1	99.6±16.7	29.1±5.6	1 <0.001	<0.001

Data are expressed as mean ± standard deviation.

GCR=cardiac rehabilitation group; GCR+IMT=cardiac rehabilitation group + inspiratory muscle training program; PEmax=maximal expiratory pressure; Pimax=maximal inspiratory pressure

* P-value derived using paired Student’s t-test, after checking for normal distributions.

** P-value derived using independent Student’s t-test for comparison of variation between the groups.

**Fig. 1:**
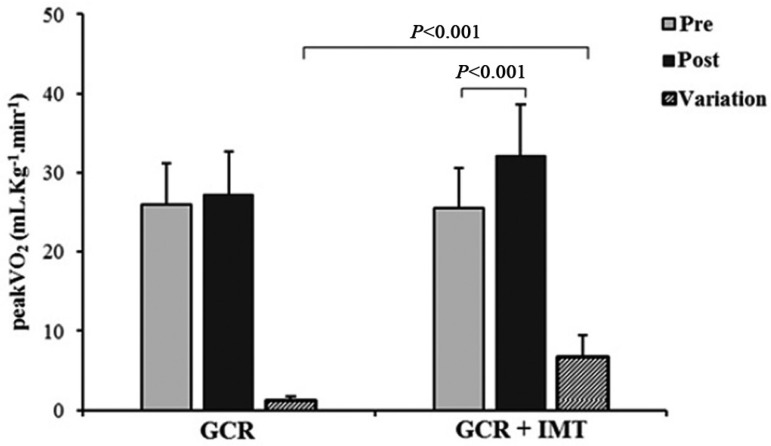
Comparison of peak oxygen consumption (peak VO_2_) between groups.

**Fig. 2:**
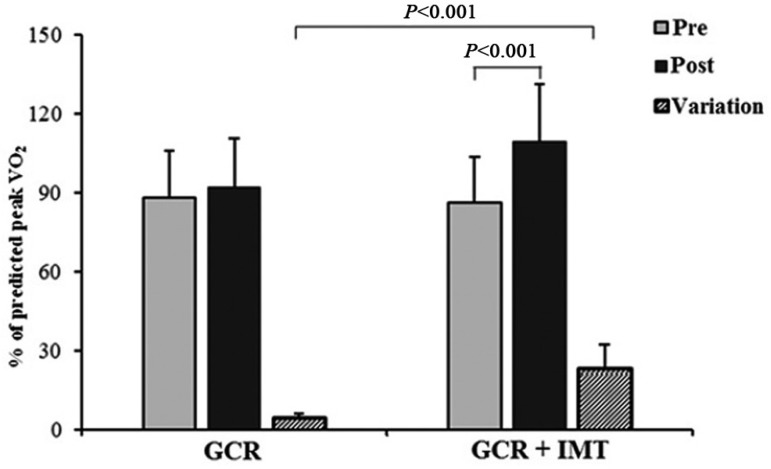
Comparison of peak oxygen consumption (% predicted) between groups.

Regarding functional capacity, it was observed that in the pre-CRP phase, the GCR group achieved approximately 88% of predicted peak VO_2_ whereas patients in the GCR+IMT achieved approximately 86% (Table 1). After intervention, patients in the GCR + IMT improved significantly their peak VO_2_. Additionally, the variation of peak VO_2_ was significantly higher in the GCR+TMI compared to the GCR, and a similar result was demonstrated for the percent-predicted peak VO_2_ (% predicted peak VO_2_) (Figures 1 and 2).

**Fig. 3:**
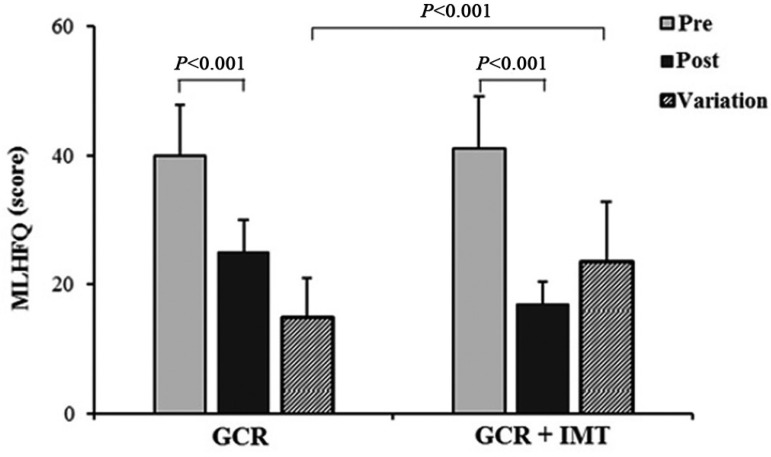
Comparison of change in QoL score, assessed with Minnesota Living with Heart Failure questionnaire (MLwHFQ), between groups.

The total MLwHFQ scores decreased significantly in both groups, indicating improvement in QoL, however, the variation in MLwHFQ scores was significantly higher in the GCR + IMT (Figure 3).

## DISCUSSION

The present study found that a short-term IMT program associated with combined aerobic and resistance training had a more pronounced effect on respiratory muscle strength, functional capacity and QoL than combined aerobic and resistance training alone in patients who underwent CABG surgery and are in phase II CRP. To the best of our knowledge, this is the first study to address the additional short-term effects of IMT associated with combined training in this patient population.

Despite the current lack of evidence demonstrating the benefits of IMT in patients who underwent CABG surgery and are in phase II CRP, it is important to mention the pioneering study of Winkelmann et al.^[18]^, which also investigated the potential additional benefits of IMT combined with aerobic training for 12 weeks, although in a different population (patients with chronic heart failure - CHF). Their study demonstrated that the addition of IMT to aerobic exercise resulted in additional improvement in PImax compared to aerobic exercise alone, and these results were similar to those reported in this study. Recently, Laoutaris et al.^[19]^, using a protocol similar to ours, showed that IMT associated with combined aerobic and resistance training in 27 patients with CHF, without inspiratory muscle weakness, is safe, and resulted in incremental benefits in PImax compared with the effects of aerobic training alone.

Studies have been conducted to evaluated the effects of combined aerobic and resistance training on functional capacity of patients undergoing CABG. Onishi et al.^[11]^ and Sumide et al.^[20]^ showed that combined training induced significant improvement in peak VO_2_ in this patient population. In contrast, Arthur et al.^[21]^, in a randomized controlled trial to compare the effect of 6 months of combined aerobic and resistance training vs aerobic training alone in women undergoing CABG, reported that after the exercise training program both groups showed statistically significant improvements in peak VO_2_.

In the present study, even over a relatively short-term period, it was observed that IMT associated with combined aerobic and resistance training provides a significant improvement in functional capacity when compared to combined training. These results indicate that the addition of IMT may have complementary effects to those obtained with combined training on functional capacity of patients who underwent CABG and are in phase II CRP. One potential explanation of this finding is that the IMT program, even over a short-term period, improves systemic vasodilation and perfusion of peripheral muscles^[22]^, promoting a more pronounced effect on functional capacity in these patients. Laoutaris et al.^[23]^ showed that addition of IMT program to aerobic training in patients with ventricular assist device resulted in an additional improvement in peak VO_2_, compared with the effects of aerobic training alone.

Regarding QoL, the values of MLwHFQ scores improved significantly in both groups. However, the IMT group showed a higher variation in MLwFQ scores. These changes may explain the additional improvement in QoL with IMT associated with combined training. Few studies have evaluated the impact of IMT associated with aerobic and resistance training on QoL specifically in patients who underwent CABG and are in phase II CRP. The addition of IMT to training programs is becoming more widespread as a potential non-pharmacological therapeutic intervention to improve QoL of patients with CHF^[18,19]^. Recently, Adamopoulos et al.^[24]^, in a 12-week prospective randomized multicenter study, have reported that IMT associated with aerobic training improves QoL in patients with CHF.

We consider that our results are relevant because even a short-term IMT program performed just twice a week with a lower inspiratory resistive loading intensity improved the variables analyzed, although it was carried out in patients without respiratory muscle weakness. Furthermore, these findings are consistent with results of previous studies conducted with patients with CHF subjected to the IMT program, though performed at higher weekly frequency and with a higher inspiratory loading intensity training protocol.

Some study limitations should be considered. First, our sample included only 24 patients submitted to CABG. However, it is of note that the number of patients who participate in phase II CRP in Brazil is extremely low. Second, an inspiratory muscle endurance testing was not performed. The third limitation is related to learning the technique of assessment of respiratory muscle strength. This test depends on the understanding and cooperation of participating individuals. Therefore, the technique can have a determinative positive effect on the outcome. This aspect can be considered as qualitatively influencing the results of the present study.

## CONCLUSION

This study demonstrates that a short-term IMT program associated with aerobic and resistance training in patients undergoing phase II of a CRP after CABG resulted in significantly large increments in respiratory muscle strength, functional capacity, and QoL. Our single-center findings also indicate that the addition of IMT, even when applied for a short period, can complement and enhance the effects of combined aerobic and resistance training and could become a simple and inexpensive adjuvant treatment, improving the efficiency of phase II cardiac rehabilitation programs within the public health system. Future large multicenter studies are needed to provide definitive proof of these benefits.

**Table:** 

**Authors’ roles & responsibilities**	
BMH	Analysis and/or interpretation of data; final approval of the manuscript; study design; implementation of projects and/ or experiments; manuscript writing or critical review of its content
DMC	Analysis and/or interpretation of data; statistical analysis; final approval of the manuscript; manuscript writing or critical review of its content
TJNG	Final approval of the manuscript; study design; implemen-tation of projects and/or experiments; manuscript writing or critical review of its content
TDS	Final approval of the manuscript; implementation of projects and/or experiments; manuscript writing or critical review of its content
MSV	Final approval of the manuscript; implementation of projects and/or experiments; manuscript writing or critical review of its content
SNP	Final approval of the manuscript; study design; manuscript writing or critical review of its content
VAB	Final approval of the manuscript; study design; manuscript writing or critical review of its content
IMA	Analysis and/or interpretation of data; Statistical analysis; final approval of the manuscript; study design; manuscript writing or critical review of its content

## References

[1] World Health Organization (2011). Global atlas on cardiovascular disease prevention and control.

[2] Brasil, Ministério da Saúde, Datasus (2010). Banco de dados do Sistema único de Saúde.

[3] Sociedade Brasileira de Cardiologia (2007). Guidelines for Unstable Angina and Non-ST-Segment Elevation
Myocardial Infarction of the Brazilian Society of Cardiology (II Edition,
2007). Arq Bras Cardiol.

[4] Smetana GW (2009). Postoperative pulmonary complications: an update on risk
assessment and reduction. Cleve Clin J Med.

[5] Borges-Santos EB, Genz IC, Longo AF, Hayashi D, Gonçalves CG, Bellinetti MB (2012). Comportamento da função pulmonar, força muscular respiratória e
qualidade de vida em pacientes submetidos às toracotomias
eletivas. Rev Col Bras Cir.

[6] Ades PA, Savage PD, Brawner CA, Lyon CE, Ehrman JK, Bunn JY (2006). Aerobic capacity in patients entering cardiac
rehabilitation. Circulation.

[7] Barros GF, Santos CS, Granado FB, Costa PT, Límaco RP, Gardenghi G (2010). Respiratory muscle training in patients submitted to coronary
arterial bypass graft. Rev Bras Cir Cardiovasc.

[8] Savci S, Degirmenci B, Saglam M, Arikan H, Inal-Ince D, Turan HN (2011). Short-term effects of inspiratory muscle training in coronary
artery bypass graft surgery: a randomized controlled trial. Scand Cardiovasc J.

[9] Ferreira PEG, Rodrigues AJ, Évora PRB (2009). Efeitos de um programa de reabilitação da musculatura
inspiratória no pós-operatório de cirurgia cardíaca. Arq Bras Cardiol.

[10] Matheus GB, Dragosavac D, Trevisan P, Costa CE, Lopes MM, Ribeiro GC (2012). Inspiratory muscle training improves tidal volume and vital
capacity after CABG surgery. Rev Bras Cir Cardiovasc.

[11] Onishi T, Shimada K, Sunayama S, Ohmura H, Sumide T, Masaki Y (2009). Effects of cardiac rehabilitation in patients with metabolic
syndrome after coronary artery bypass grafting. J Cardiol.

[12] Mahler DA, American College of Sports Medicine (1995). ACSM's guidelines for exercise testing and prescription,.

[13] Dall'Ago P, Chiappa GR, Guths H, Stein R, Ribeiro JP (2006). Inspiratory muscle training in patients with heart failure and
inspiratory muscle weakness: a randomized trial. J Am Coll Cardiol.

[14] American Thoracic Society/European Respiratory Society (2002). ATS/ERS Statement on respiratory muscle testing. Am J Resp Crit Care Med.

[15] Neder JA, Andreoni S, Lerario MC, Nery LE (1999). Reference values for lung function tests. II. Maximal respiratory
pressures and voluntary ventilation. Braz J Med Biol Res.

[16] Meneghelo RS, Araújo CGS, Stein R, Mastrocolla LE, Albuquerque PF, Serra SM, Sociedade Brasileira de Cardiologia (2010). III Diretrizes sobre teste ergométrico. Arq Bras Cardiol.

[17] Carvalho VO, Guimarães GV, Carrara D, Bacal F, Bocchi EA (2009). Validação da versão em português do Minnesota Living with Heart
Failure Questionnaire. Arq Bras Cardiol.

[18] Winkelmann ER, Chiappa GR, Lima CO, Viecili PR, Stein R, Ribeiro JP (2009). Addition of inspiratory muscle training to aerobic training
improves cardiorespiratory responses to exercise in patients with heart
failure and inspiratory muscle weakness. Am Heart J.

[19] Laoutaris ID, Adamopoulos S, Manginas A, Panagiotakos DB, Kallistratos MS, Doulaptsis C (2013). Benefits of combined aerobic/resistance/inspiratory training in
patients with chronic heart failure. A complete exercise model? A
prospective randomised study. Int J Cardiol.

[20] Sumide T, Shimada K, Ohmura H, Onishi T, Kawakami K, Masaki Y (2009). Relationship between exercise tolerance and muscle strength
following cardiac rehabilitation: comparison of patients after cardiac
surgery and patients with myocardial infarction. J Cardiol.

[21] Arthur HM, Gunn E, Thorpe KE, Ginis KM, Mataseje L, McCartney N (2007). Effect of aerobic vs combined aerobic-strength training on
1-year, post-cardiac rehabilitation outcomes in women after a cardiac
event. J Rehabil Med.

[22] Chiappa GR, Roseguini BT, Vieira PJ, Alves CN, Tavares A, Winkelmann ER (2008). Inspiratory muscle training improves blood flow to resting and
exercising limbs in patients with chronic heart failure. J Am Coll Cardiol.

[23] Laoutaris ID, Dritsas A, Adamopoulos S, Manginas A, Gouziouta A, Kallistratos MS (2011). Benefits of physical training on exercise capacity, inspiratory
muscle function, and quality of life in patients with ventricular assist
devices long-term postimplantation. Eur J Cardiovasc Prev Rehabil.

[24] Adamopoulos S, Schmid J, Dendale P, Poerschke D, Hansen D, Dritsas A (2014). Combined aerobic/inspiratory muscle training vs. aerobic training
in patients with chronic heart failure: The Vent-HeFT trial: a European
prospective multicentre randomized trial. Eur J Heart Fail.

